# The Wearing Away of Teeth

**Published:** 1890-10

**Authors:** 


### The Wearing Away of Teeth.

Mr. MacLeod, at a meeting of the Odonto-Chirurgical Society said, according to the *Lancet*, that having his attention drawn by a single case, he had been led to examine the teeth of various bag-pipers, and all of them represented wearing away of the cutting edges of the six front teeth, in a greater or lesser degree, varying with the density of the tooth structure and the time engaged in pipe-playing. He found on inquiry, that on the average it took about four years to make a well-marked impression, but that once the enamel edge was worn through, the wearing away was more rapid. Every one was aware of the way in which the tobacco-pipe wore the teeth of the smoker, but this was not to be wondered at, the baked pipe-clay being a hard and gritty substance, but that the horn mouth-piece should have such appreciable effect, was, he thought, a matter of curious interest. He mentioned, however, that the mouth-pieces suffered more than the teeth, the average life of a horn mouth-piece being twelve to eighteen months, that of a bone or ivory one being about two years. The peculiarity noticed was a crescent shaped aperture on the cutting edge of the front teeth in three localities, namely: between the central incisors, and between the lateral and canine on both sides.

THE DEADLY COLD BED.—A writer in the *Good Housekeeping* says: "If trustworthy statistics could be had of the number of persons who die every year, or become permanently diseased from sleeping in damp or cold beds, they would probably be astonishing and appalling. It is a peril that constantly besets traveling men, and it they are wise, they will invariably insist on having their beds aired and dried, even at the risk of causing much trouble to their landlords. But the peril resides in the home, and the cold "spare room" has slain its thousands of hapless guests, and will go on with its slaughter till people learn wisdom. Not only the guest, but the family often suffer the penalty of sleeping in cold rooms, and chilling their bodies at a time when they need all their bodily heat, by getting between cold sheets. Even in warm summer weather, a cold, damp bed will get in its deadly work. It is a needless peril, and the neglect to provide dry rooms and beds has in it the elements of murder and suicide."—*Druggists' Circular.*

